# Transcutaneous electrical acupoint stimulation for preventing postoperative nausea and vomiting in patients undergoing breast surgery: a systematic review and meta-analysis of randomized controlled trials

**DOI:** 10.3389/fmed.2026.1830545

**Published:** 2026-06-03

**Authors:** Jing Zhao, Yue-zong Lv, Li Xue, Qi-hong Shen

**Affiliations:** 1 Department of Anesthesiology, Jiashan First People's Hospital, Jiaxing, China; 2 Department of Anesthesiology, Affiliated Hospital of Jiaxing University, Jiaxing, China; 3 Department of Breast Disease, Affiliated Hospital of Jiaxing University, Jiaxing, China

**Keywords:** analgesia, breast cancer surgery, meta-analysis, postoperative nausea and vomiting, quality of recovery, transcutaneous electrical acupoint stimulation

## Abstract

**Background:**

Postoperative nausea and vomiting (PONV) remains a frequent challenge for patients undergoing breast cancer surgery. Transcutaneous electrical acupoint stimulation (TEAS) is increasingly utilized as a needle-free, neuromodulatory adjunct in perioperative care. Although a prior meta-analysis evaluated TEAS for PONV across general surgical populations, breast surgery-specific evidence remains lacking. This meta-analysis evaluates the efficacy of TEAS for preventing PONV and enhancing early recovery in this specific surgical population.

**Methods:**

We systematically searched PubMed, Embase, Cochrane Library, Web of Science, and three Chinese databases (CNKI, VIP, Wanfang) for randomized controlled trials (RCTs) comparing perioperative TEAS with sham stimulation or routine care, up to March 10, 2026. The primary outcome was overall PONV incidence. Secondary outcomes included postoperative nausea, vomiting, pain scores, and early recovery quality (QoR). Data were synthesized using RevMan 5.4, and the certainty of evidence was assessed with GRADE.

**Results:**

Fifteen RCTs comprising 1,752 patients were included. TEAS significantly reduced overall PONV risk [RR = 0.61, 95% CI (0.49, 0.77), *P* < 0.01] with no heterogeneity (I^2^ = 0%). Patients receiving TEAS also experienced lower postoperative nausea, vomiting, pain scores, reduced need for rescue antiemetics, and improved early QoR scores.

**Conclusion:**

TEAS might be an effective and non-pharmacological adjunct for reducing PONV and supporting early recovery in breast cancer surgery. These findings support its integration into perioperative care, although further large-scale, sham-controlled RCTs are warranted to establish standardized protocols.

**Systematic review registration:**

https://www.crd.york.ac.uk/PROSPERO/view/CRD420261333755.

## Introduction

Breast cancer surgery is frequently associated with postoperative nausea and vomiting (PONV). Without prophylaxis, PONV incidence ranges from 40 to 60% due to female gender, volatile anesthetics, and postoperative opioids (1, 2). Severe PONV and uncontrolled pain can lead to wound dehiscence, electrolyte imbalances, and delayed hospital discharge, increasing healthcare costs and reducing patient satisfaction (3, 4).

Standard pharmacological prophylaxis using 5-HT3 receptor antagonists, corticosteroids, and dopamine receptor antagonists can cause side effects such as headaches, dizziness, and prolonged QT intervals (5, 6). Furthermore, reliance on opioids for postoperative analgesia exacerbates PONV risk. Consequently, non-pharmacological adjunctive therapies are gaining interest within Enhanced Recovery After Surgery (ERAS) protocols to optimize outcomes with fewer side effects.

Transcutaneous electrical acupoint stimulation (TEAS) combines traditional Chinese acupuncture principles with transcutaneous electrical nerve stimulation (TENS). By applying electrical currents to specific acupoints (such as PC6, ST36), TEAS stimulates sensory nerve fibers and regulates the autonomic nervous system (7, 8). It promotes endogenous opioid release and modulates neurotransmitters involved in the emetic reflex. Compared to manual acupuncture, TEAS is non-invasive, easier to standardize, and carries no risk of needle-related infections.

Although TEAS has been evaluated for PONV prevention in general surgical populations (9), high-quality evidence specific to breast surgery is lacking. Chen et al.'s meta-analysis (9) included mixed surgical types (laparoscopic, gynecologic, orthopedic). Patients undergoing breast cancer surgery differ fundamentally: they are almost exclusively female, receive volatile anesthetics and opioids, and have higher baseline PONV rates, making them a distinct high-risk population (10, 11). Moreover, prior reviews focused narrowly on PONV incidence, without evaluating holistic recovery outcomes such as quality of recovery (QoR). Several RCTs have evaluated TEAS for PONV in breast surgery, but variations in sample sizes and stimulation parameters have yielded inconsistent conclusions (12–14). This meta-analysis evaluates the impact of perioperative TEAS on PONV, postoperative pain, and recovery quality in breast cancer surgery to provide updated evidence for clinical practice.

## Methods

### Study protocol and registration

This systematic review and meta-analysis was conducted following PRISMA guidelines. The protocol was registered in PROSPERO (CRD420261333755) before the start of the study.

### Search strategy

We searched PubMed, Embase, Cochrane Library, Web of Science, and three Chinese databases (CNKI, Wanfang, VIP) from inception to March 10, 2026. The search combined both controlled vocabulary (MeSH terms) and free-text keywords for “transcutaneous electrical acupoint stimulation,” “breast surgery,” and “postoperative nausea and vomiting.” We restricted the search to RCTs involving adult human participants (aged 18 years and older). No language restrictions were applied. For any non-English or non-Chinese articles identified, titles and abstracts were screened using Google Translate; full texts were translated with the assistance of a bilingual reviewer if potentially eligible. References of included studies and relevant reviews were manually screened to identify additional eligible trials. We also searched ClinicalTrials.gov and the WHO International Clinical Trials Registry Platform (ICTRP) for ongoing or unpublished trials. The full search strategies for all databases are included in the Supplementary Material.

### Inclusion and exclusion criteria

Study eligibility was strictly determined based on the PICOS framework: (1) Population (P): Adult female patients (aged 18 years and older) scheduled for elective breast cancer surgery under general anesthesia. Both mastectomy and breast-conserving surgery were included, as the study aimed to evaluate TEAS across the spectrum of breast cancer surgeries. (2) Intervention (I): Perioperative application of TEAS, regardless of the specific acupoint combinations, stimulation parameters (frequency, intensity), or the timing of the intervention. (3) Comparison (C): Patients receiving either sham TEAS (electrode application at the same acupoints without electrical output) or standard perioperative care. (4) Outcomes (O): The primary outcome was the incidence of overall PONV. Secondary outcomes included the incidence of postoperative nausea (PON), the incidence of postoperative vomiting (POV), the requirement for rescue antiemetics, postoperative pain intensity measured by the Visual Analog Scale (VAS) or Numeric Rating Scale (NRS), and the quality of recovery evaluated by validated questionnaires (QoR-15 or QoR-40). Overall PONV was defined as the occurrence of nausea and vomiting within the first 48 h postoperatively, or the longest reported period if multiple windows were provided. (5) Study Design (S): Randomized controlled trials (RCTs).

Exclusion criteria were as follows: (1) non-randomized designs, including observational studies, retrospective cohorts, case reports, reviews, and animal studies; (2) interventions involving invasive acupuncture techniques, such as manual acupuncture or electroacupuncture with needles; (3) studies focusing exclusively on cosmetic breast augmentation; and (4) duplicate publications or articles with incomplete outcome data that could not be resolved by contacting the original authors.

### Data extraction and risk of bias assessment

Two reviewers independently screened the records and extracted data into a predefined spreadsheet. Based on the characteristics of the included studies, the extracted items primarily included: (1) basic study information (first author and publication year); (2) participant demographics (sample size in the TEAS and control groups, age); (3) intervention details (acupoints selected, stimulation frequency such as 2 Hz or 2/100 Hz, intervention timing, and duration); (4) control group types (sham TEAS or routine care); and (5) outcome measures (incidence of overall PONV, PON, POV, rescue antiemetic requirements, postoperative pain scores, and QoR scores). Any discrepancies during the extraction process were resolved through discussion with a third reviewer.

The methodological quality of the included RCTs was independently evaluated using the Cochrane Risk of Bias 2 (RoB 2) tool. The assessment covered five specific domains: bias arising from the randomization process, bias due to deviations from intended interventions, bias due to missing outcome data, bias in measurement of the outcome, and bias in selection of the reported result. Any disagreements in the quality assessment were resolved by consensus or consultation with the third reviewer.

Inter-rater agreement for study selection and risk of bias assessment was calculated using Cohen's kappa. The kappa values were 0.92 for study inclusion and 0.86 for RoB 2 domain judgments, indicating excellent agreement.

### Statistical analysis

All meta-analyses were conducted using Review Manager software (RevMan, version 5.4; The Cochrane Collaboration). For dichotomous outcomes (incidences of overall PONV, PON, POV, and the use of rescue antiemetics), effect sizes were expressed as risk ratios (RRs) with 95% confidence intervals (CIs). For continuous outcomes, mean differences (MDs) with 95% CIs were calculated when studies utilized the same measurement scales. Standardized mean differences (SMDs) with 95% CIs were applied when different psychometric scales were used to assess the same outcome. Given the inherent clinical heterogeneity across the included trials regarding specific TEAS parameters (such as stimulation frequency, intensity, and duration) and anesthetic management, a random-effects model was universally adopted for all pooled analyses to provide more conservative and generalizable estimates. Specifically, the DerSimonian-Laird (DL) method (the default estimator in RevMan 5.4) was used to estimate the between-study variance (τ^2^). Statistical heterogeneity among the included studies was evaluated using the Cochran Q test and quantified by the I-squared (I^2^) statistic, with an I^2^ value > 50% indicating substantial heterogeneity. An overall effect size was considered statistically significant at a two-sided *P*-value < 0.05. We performed subgroup analyses by control type (sham vs. routine care) to assess potential expectation bias.

To verify the stability of the pooled results and investigate potential sources of high heterogeneity, sensitivity analyses were performed by sequentially omitting one study at a time (the leave-one-out method). Potential publication bias was evaluated through visual inspection of funnel plot asymmetry. Quantitative tests for funnel plot asymmetry (Egger's test) were not performed, as the number of included trials for each outcome was fewer than 10, in strict accordance with the recommendations of the Cochrane Handbook. A conventional threshold of P < 0.05 was considered indicative of statistically significant publication bias. Finally, the overall certainty of evidence for each main outcome was evaluated using the Grading of Recommendations Assessment, Development and Evaluation (GRADE) approach. The evidence quality was rated as “High,” “Moderate,” “Low,” or “Very Low” by assessing five critical domains: risk of bias, inconsistency, indirectness, imprecision, and publication bias.

## Results

### Study selection and characteristics

**Figure 1:**
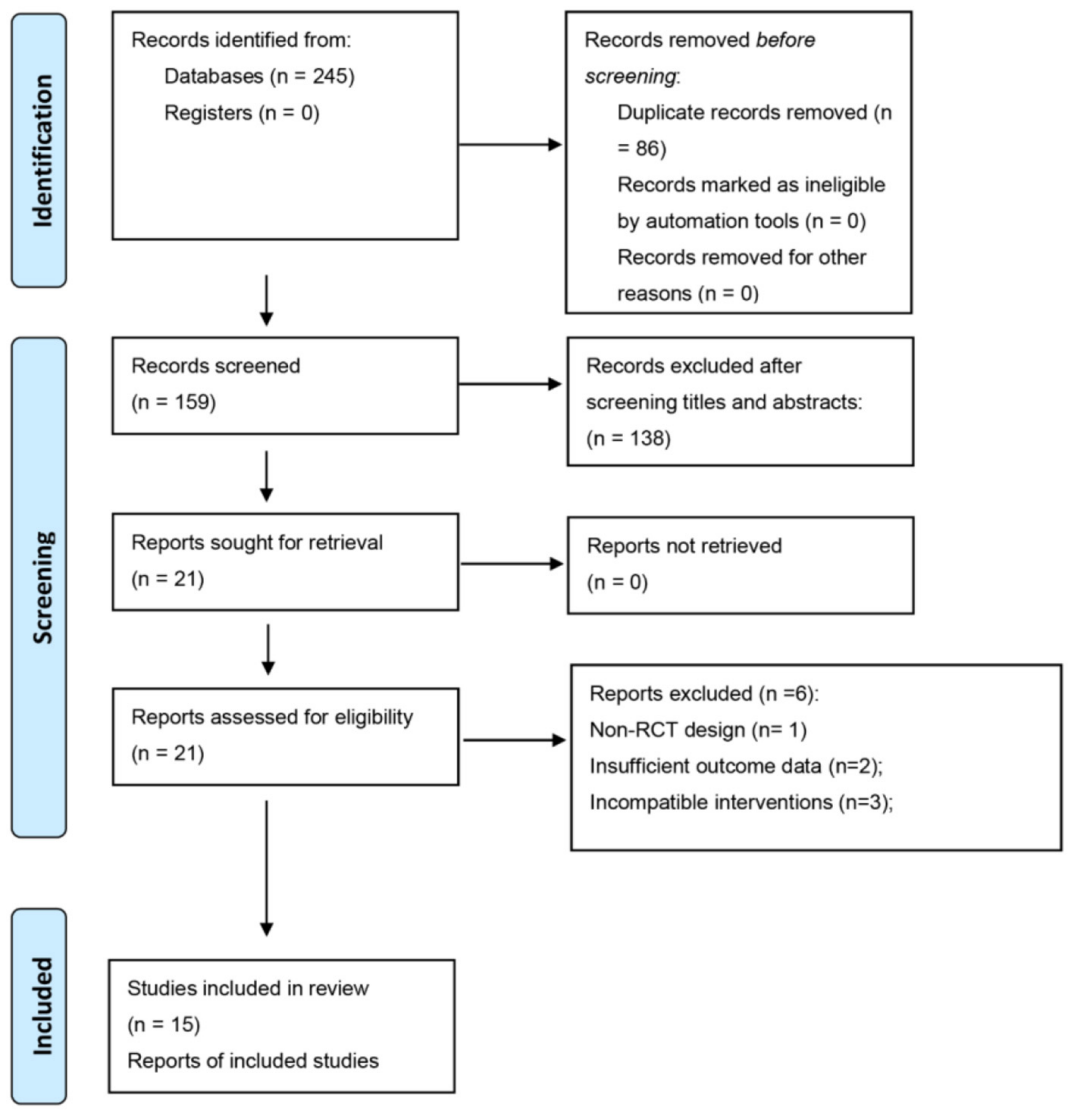
PRISMA flow diagram of the study selection process.

The initial literature search yielded a total of 245 records. After removing 86 duplicates, the titles and abstracts of 159 unique records were screened, resulting in the exclusion of 138 irrelevant articles. The full texts of the remaining 21 potentially eligible studies were then assessed in detail. Of these, 6 articles were excluded for reasons such as non-RCT design, incompatible interventions, or insufficient outcome data. Ultimately, 15 RCTs were included in the meta-analysis (12–26). Compared with the prior TEAS meta-analysis (9) across various cancer types, 11 of the 15 trials in our analysis have not been previously synthesized, including three trials published after its search date. This substantial amount of new breast-specific evidence justifies an updated synthesis. The detailed study selection process is illustrated in the PRISMA flow diagram (Figure 1).

The 15 included trials encompassed a total of 1,752 patients undergoing breast cancer surgery, with 980 assigned to the TEAS group and 772 to the control group. The intervention protocols varied across the studies: the Neiguan (PC6) acupoint was the most frequently targeted site, and stimulation frequencies primarily consisted of constant low frequency (2 Hz) or alternating dense-disperse frequencies (2/100 Hz or 2/30 Hz). For the comparison groups, five studies utilized sham TEAS with no electrical output (15, 19, 20, 24, 25), whereas the remaining 10 studies employed standard routine care. Detailed baseline characteristics, surgical types, and specific TEAS parameters for each trial are summarized in Tables 1a, b.

**Table 1a:** General characteristics of the included studies.

Study (year)	Sample size (E/C)	Age (years, E/C, mean ±SD or range)	BMI (kg/m^2^, E/C, mean ±SD or range)	ASA (I/II, E/C)	Type of surgery	Included outcomes
Ao L (2021)	32/33	45.6 ± 9.8/46.9 ± 8.6	23.4 ± 4.2/22.6 ± 3.3	26/6 vs. 24/9	Radical mastectomy	Total PONV, pain
Lu Z (2021)	388/188	48.2 ± 9.0, 48.2 ± 9.1/48.2 ± 8.2	23.6 ± 2.5, 23.7 ± 2.6/23.3 ± 2.8	144/52,135/53 vs. 135/49	Mastectomy	Nausea, vomiting
Luo L (2024)	66/66	49.1 ± 10.2/49.4 ± 9.7	22.8 ± 3.3/22.4 ± 2.5	31/35 vs. 30/36	Mastectomy	Nausea, vomiting
Zhang M (2025)	33/33	50.5 ± 6.4/50.2 ± 5.3	24.2 ± 2.4/24.3 ± 2.3	31/35 vs. 30/36	Modified radical mastectomy	Nausea, vomiting, pain, QoR
Yan L (2025)	70/68	48 ± 8/48 ± 8	24.1 ± 2.2/23.8 ± 2.5	52/18 vs. 48/20	Modified radical mastectomy	Total PONV, pain
Meng J (2023)	60/60	47.4 ± 5.4/47.3 ± 5.1	23.3 ± 1.6/23.2 ± 1.7	20/40 vs. 22/38	Radical mastectomy	Total PONV, pain
Zhang A (2021)	70/70	45.9 ± 5.6/45.6 ± 5.5	23.8 ± 2.6/23.6 ± 2.6	33/37 vs. 29/41	Breast cancer surgery	Total PONV, pain
Jin W (2020)	30/31	48.2 ± 6.9/50.9 ± 7.1	22.9 ± 2.2/23.9 ± 3.2	9/21 vs. 8/23	Breast cancer surgery	Total PONV, QoR
Sun H (2020)	20/20	44.2 ± 5.2/44.2 ± 5.2	Not reported	Not reported	Modified radical mastectomy	Nausea, vomiting, pain
Mao H (2019)	40/40	35–55 (range)	19.9–26.7 (range)	I or II (no details)	Breast cancer surgery	Nausea, vomiting, pain
Zhou W (2017)	30/30	31–67 (range)	Not reported	I or II (no details)	Modified radical mastectomy	Nausea, vomiting, pain
Wang Z (2017)	34/34	34 ± 7/36 ± 9	Weight: 54 ± 7/56 ± 8 kg	31/3 vs. 30/4	Breast cancer surgery	Total PONV, QoR
Hu X (2014)	30/30	30–60 (range)	Weight: 50–80 kg	I-II (no details)	Radical mastectomy	Total PONV, pain
Liu Y (2011)	47/39	52/50 (mean)	Weight: 56 ± 5/57 ± 5 kg	I-II (no details)	Breast cancer surgery	Nausea, vomiting, pain
Yu J (2010)	30/30	20–70 (range)	Weight: 45–70 kg	I-II (no details)	Radical mastectomy	Total PONV, pain

E/C, Experimental group; (TEAS), Control group; ASA, American Society of Anesthesiologists; BMI, Body mass index; PONV, Postoperative nausea and vomiting; QoR, Quality of recovery (assessed via QoR-15 or QoR-40 questionnaires).

**Table 1b:** Intervention parameters of the included studies.

Study (year)	Control intervention	Acupoints selected	TEAS timing and duration	Freq (Hz)	Anesthetic regimen	Baseline PONV prophylaxis
Ao L (2021)	Sham TEAS	LI4, PC6, ST36	Preop 30 min; Postop: 4h,12h on D0, then 3x/day on D1-2 (30 min each)	2/100	Propofol, remifentanil, sufentanil, cisatracurium; BIS 40–60	None reported
Lu Z (2021)	Sham TEAS	PC6, CV17	Preop 30 min (single session)	2/10	Propofol, remifentanil, fentanyl; TIVA	None reported
Luo L (2024)	Routine care	PC6	Preop 30 min until PACU discharge	2/100	Sevoflurane (2%−3%), remifentanil, sufentanil, rocuronium	Dexamethasone 8 mg
Zhang M (2025)	Sham TEAS	PC6, LI4, ST36, LR3	Preop 30 min until end of surgery	2/100	Propofol, remifentanil, sufentanil, cisatracurium	Tropisetron 6 mg
Yan L (2025)	Sham TEAS	PC6, ST36, CV17	Preop 30 min to end of surgery; plus POD1-3 (30 min daily)	2/100	Propofol, remifentanil, sufentanil, rocuronium; BIS 40–60	None reported
Meng J (2023)	Routine care	LI4, PC6, ST36	Preop 30 min only	2/30	Propofol, remifentanil, sufentanil, cisatracurium	None reported
Zhang A (2021)	Routine care	LI4, PC6, ST36	Preop 30 min only	2/30	Propofol TCI, sufentanil, cisatracurium	None reported
Jin W (2020)	Routine care	LI4, PC6, ST36, SP6	Preop 30 min until end of surgery	2/100	Propofol, remifentanil, sufentanil, cisatracurium	Palonosetron 0.25 mg
Sun H (2020)	Routine care	PC6, ST36	Preop 30 min to end of surgery; plus POD1-3 (30 min daily)	2/100	Propofol, fentanyl, vecuronium; sevoflurane	None reported
Mao H (2019)	Routine care	Routine acupoints	Preop 10 min to end of surgery	2/100	Propofol, remifentanil, sufentanil, rocuronium; BIS 40–60	Tropisetron 5 mg in PCA
Zhou W (2017)	Routine care	PC6, LI4, ST36	Preop 30 min to end of surgery	2	Propofol, remifentanil, sufentanil, rocuronium; sevoflurane	None reported
Wang Z (2017)	Routine care	LI4, PC6, ST36	Preop 30 min only	2/10	Propofol, remifentanil, sufentanil, cisatracurium; laryngeal mask, BIS 40–60	Tropisetron 5 mg
Hu X (2014)	routine care	LI4, PC6, ST36	Preop 30 min only	2/30	Propofol TCI, fentanyl, vecuronium	Tropisetron 2 mg
Liu Y (2011)	Sham TEAS	PC6	Preop 30 min only	2–100	Epidural (lidocaine+ropivacaine) + midazolam	None reported
Yu J (2010)	Routine care	LI4, PC8, PC6, TE5	Preop 30 min until end of surgery	2/100	Propofol TCI, sufentanil, cisatracurium; remifentanil infusion	None reported

TEAS, Transcutaneous electrical acupoint stimulation; Sham TEAS, Sham transcutaneous electrical acupoint stimulation (electrodes placed without electrical output, or minimal output to mimic skin sensation without therapeutic effect); Routine Care, Conventional standard perioperative care or general anesthesia without any acupoint electrical stimulation; Freq (Hz), Frequency of transcutaneous electrical acupoint stimulation (in Hertz); PONV, Postoperative nausea and vomiting; QoR, Quality of recovery (assessed via QoR-15 or QoR-40 questionnaires); PC6, Neiguan (Pericardium 6); LI4, Hegu (Large Intestine 4); ST36, Zusanli (Stomach 36); CV17, Danzhong (Conception Vessel 17); LR3, Taichong (Liver 3); SP6, Sanyinjiao (Spleen 6); PC8, Laogong (Pericardium 8); TE5, Waiguan (Triple Energizer 5); BIS, bispectral index; PACU, post-anesthesia care unit; PCA, patient-controlled analgesia; POD, postoperative day; TCI, target-controlled infusion; TIVA, total intravenous anesthesia.

### Risk of bias assessment

**Figure 2:**
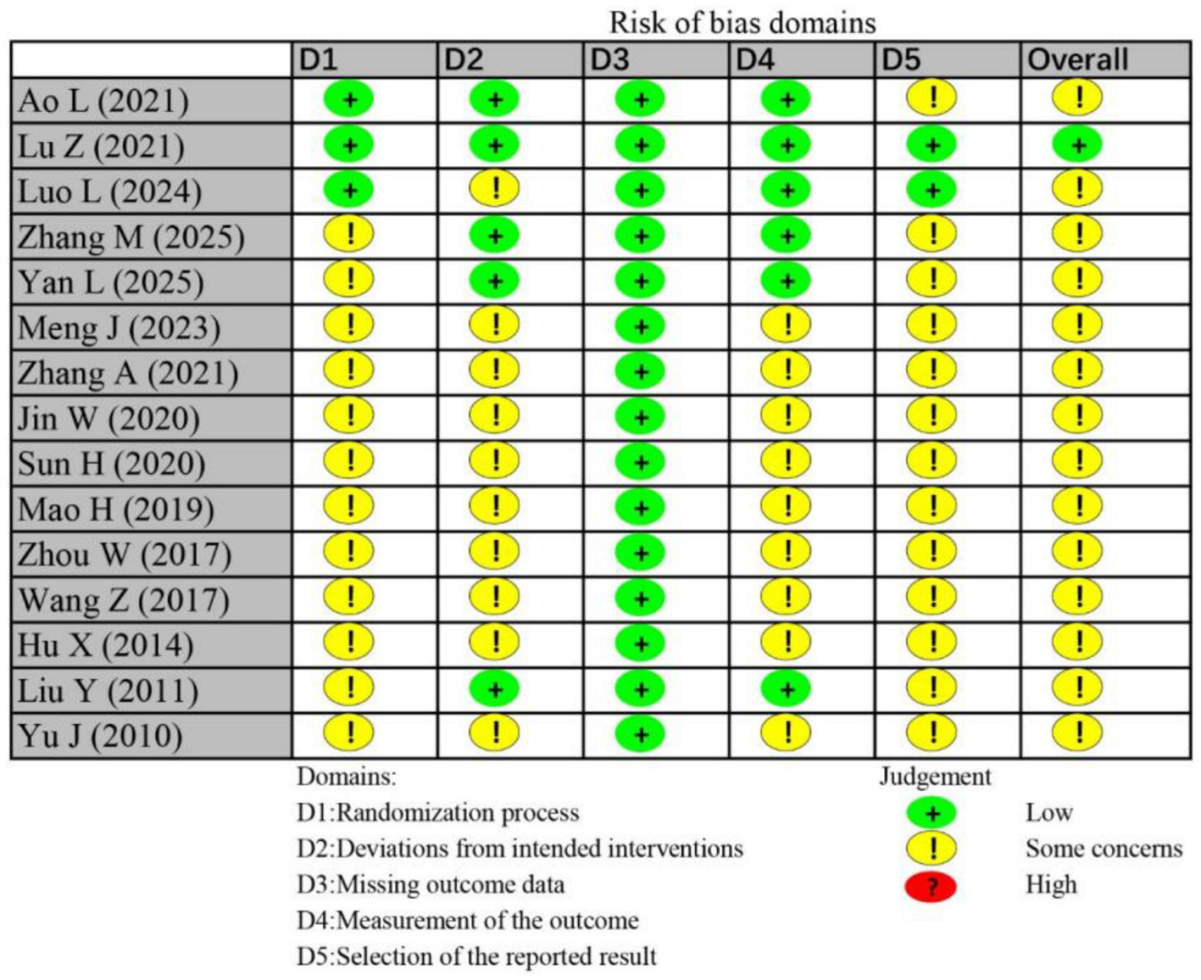
Risk of bias summary for the included randomized controlled trials.

Based on the RoB 2 assessment, one study (19) had an overall “low risk” of bias, while the other 14 had “some concerns.” In Domain 1, three studies (14, 15, 19) reported adequate allocation concealment (“low risk”). In Domain 2, five studies(15, 19, 20, 24, 25) using sham TEAS achieved strict blinding (“low risk”), whereas 10 unblinded trials using standard care raised “some concerns” regarding potential performance bias. All trials had complete outcome data (“low risk” in Domain 3). For Domain 4, the subjective nature of the outcomes combined with unclear assessor blinding led to “some concerns” in 10 studies. Finally, 13 studies lacked public prospective trial registrations (“some concerns” in Domain 5). The detailed risk of bias summary is presented in Figure 2.

### Primary outcome: incidence of overall PONV

**Figure 3:**
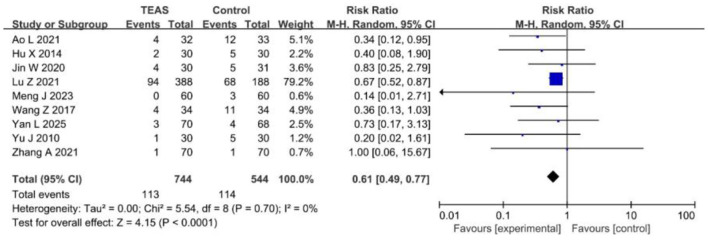
Forest plot comparing the incidence of overall postoperative nausea and vomiting (PONV) between the TEAS and control groups.

Nine studies reported the incidence of overall PONV. The pooled analysis demonstrated that perioperative TEAS significantly reduced the risk of overall PONV compared to the control group [RR = 0.61, 95% CI (0.49, 0.77), *P* < 0.01], with no evidence of statistical heterogeneity across the included trials (I^2^ = 0%, τ^2^ = 0.00) (Figure 3). The time windows for PONV assessment varied across the nine trials: three reported within 48 h (15, 23, 24), five within 24 h (12, 18, 19, 22, 26), and one within 12 h (13). None of the trials distinguished early (0–6 h) vs. late (6–24 h) PONV. Subgroup analyses based on control type showed consistent results. In sham-controlled trials (Ao et al., Lu et al., Yan et al.), TEAS significantly reduced the incidence of PONV [RR = 0.65, 95% CI (0.50, 0.83), I^2^ = 0%, τ^2^ = 0.00]. In routine care-controlled trials (Hu et al., Jin et al., Meng et al., Wang et al., Yu et al., Zhang et al.), a similar effect was observed [RR = 0.44, 95% CI (0.23, 0.83), I^2^ = 0%, τ^2^ = 0.00, Supplement figure].

### Secondary outcomes: PON, POV, and rescue antiemetics

**Figure 4:**
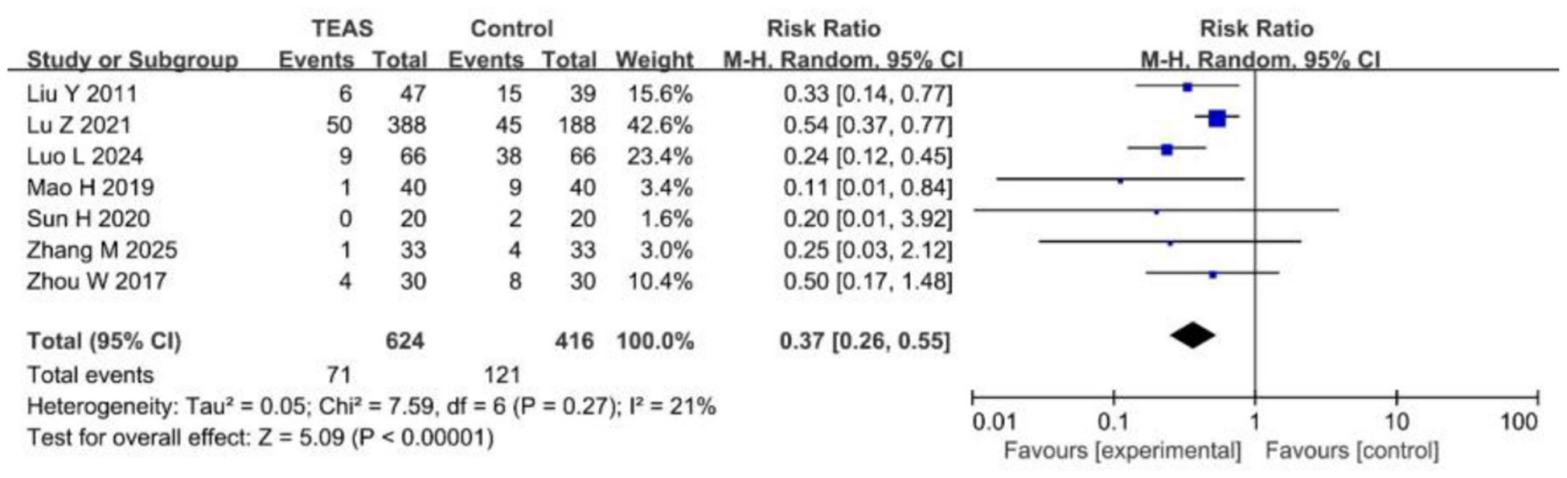
Forest plot comparing the incidence of isolated postoperative nausea (PON) between the TEAS and control groups.

**Figure 5:**
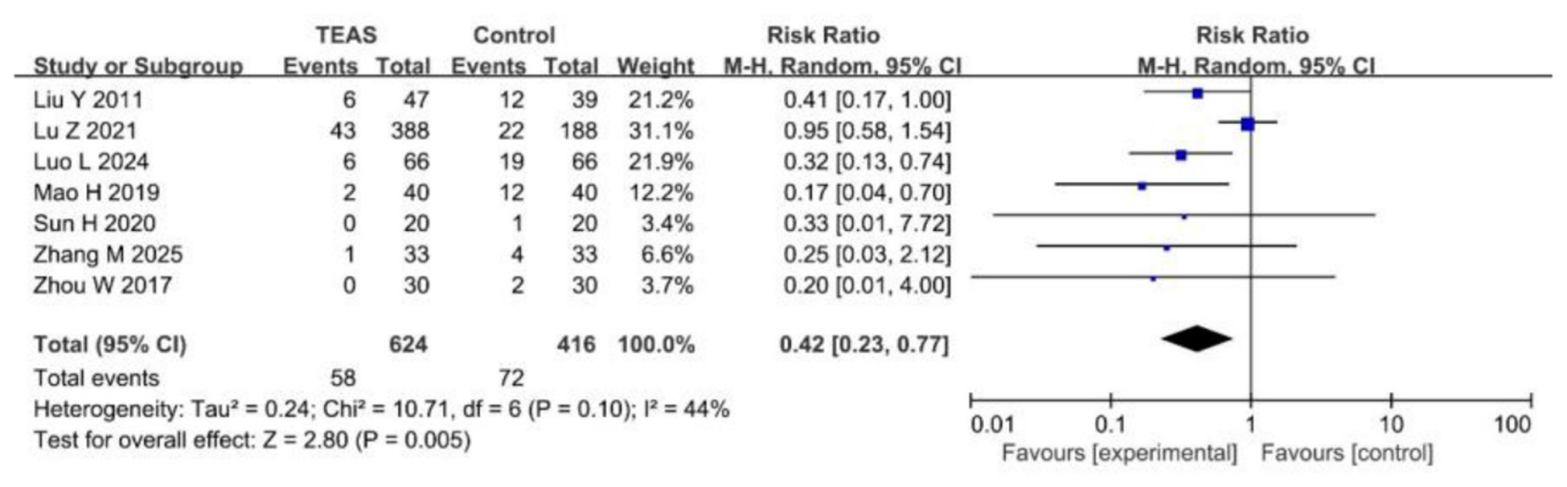
Forest plot comparing the incidence of isolated postoperative vomiting (POV) between the TEAS and control groups.

**Figure 6:**
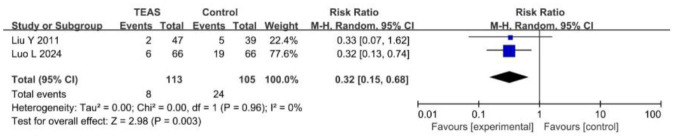
Forest plot comparing the requirement for rescue antiemetic medication between the TEAS and control groups.

Data for PON and POV were available in seven studies, respectively. Consistent with the primary outcome, TEAS significantly decreased the risk of both PON [RR = 0.37, 95% CI (0.26, 0.55), *P* < 0.01; I^2^ = 21%, Figure 4, τ^2^ = 0.05] and POV [RR = 0.42, 95% CI (0.23, 0.77), *P* < 0.01; I^2^ = 44%, Figure 5, τ^2^ = 0.24]. Consequently, the requirement for rescue antiemetic medication was also significantly lower in the TEAS group [two studies; RR = 0.32, 95% CI (0.15, 0.68), *P* < 0.01; I^2^ = 0%, τ^2^ = 0.00, Figure 6]. Regarding safety, no severe adverse events related to the TEAS intervention (such as localized skin burns or infections) were reported in any of the included trials.

### Secondary outcomes: postoperative pain scores and quality of recovery

**Figure 7:**
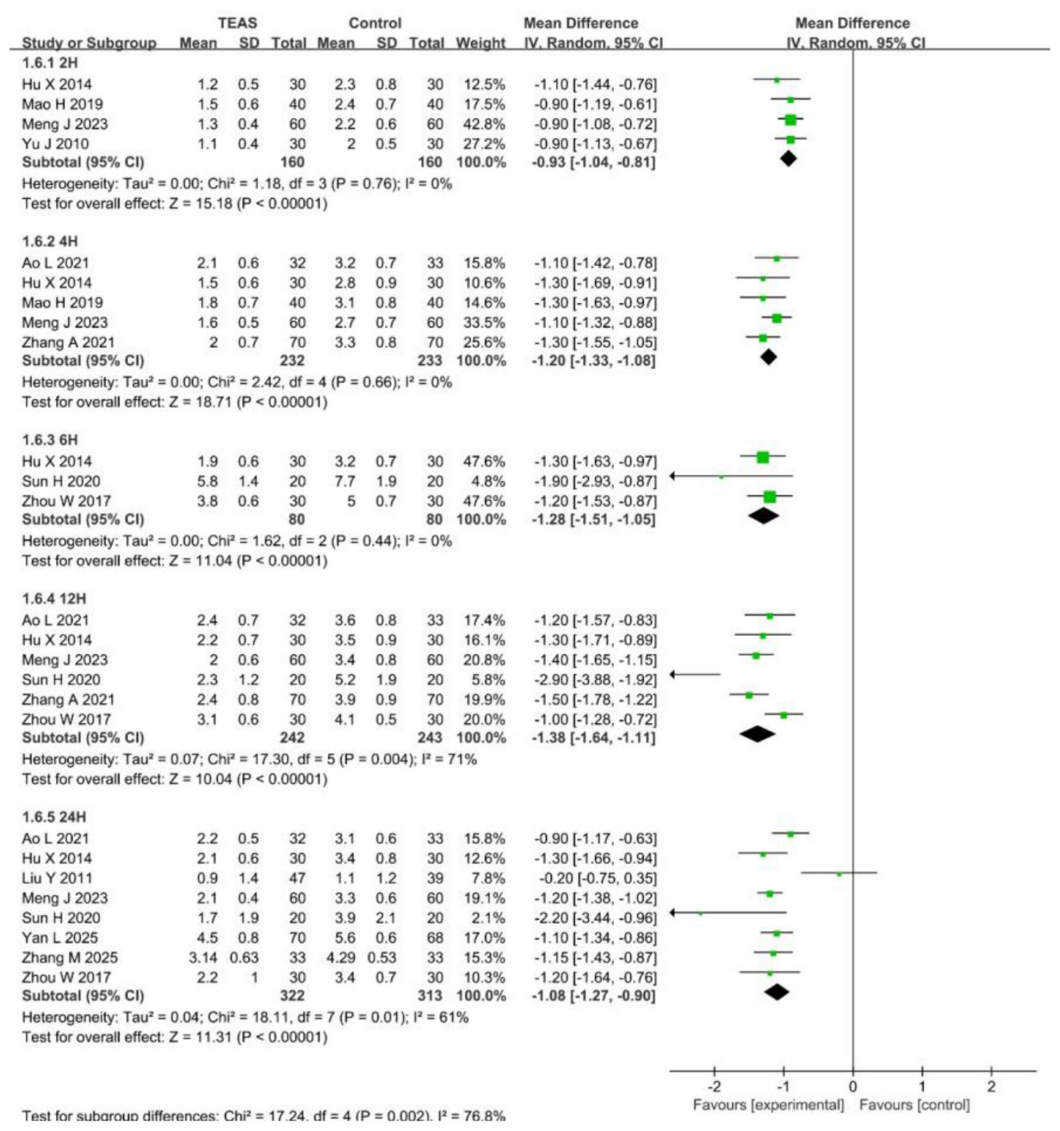
Forest plot of subgroup analysis for postoperative pain scores across multiple time points (2, 4, 6, 12, and 24 h).

We further analyzed postoperative pain scores at each reported time point. Compared with control, TEAS significantly reduced pain scores at 2 h [MD = −0.93, 95% CI (−1.04, −0.81), I^2^ = 0%, τ^2^ = 0.00], 4 h [MD = −1.20, 95% CI (−1.33, −1.08), I^2^ = 0%, τ^2^ = 0.00], 6 h [MD = −1.28, 95% CI (−1.51, −1.05), I^2^ = 0%, τ^2^ = 0.00], 12 h [MD = −1.38, 95% CI (−1.64, −1.11), I^2^ = 71%, τ^2^ = 0.07], and 24 h [MD = −1.08, 95% CI (−1.27, −0.90), I^2^ = 61%, τ^2^ = 0.04, Figure 7]. The analgesic effect of TEAS was consistent in the early postoperative period (2–6 h) with no statistical heterogeneity, whereas substantial heterogeneity emerged at 12 and 24 h.

**Figure 8:**
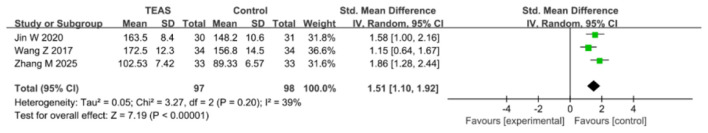
Forest plot comparing the early Quality of Recovery (QoR) scores between the TEAS and control groups.

Three studies assessed the early quality of recovery using validated QoR questionnaires. Specifically, Wang et al. (26) and Jin et al. (22) used QoR-40, while Zhang et al. (20) used QoR-15. We used SMD due to variation in scoring scales (QoR-15 vs. QoR-40). The pooled analysis indicated a significant improvement in QoR scores for patients receiving TEAS [SMD = 1.51, 95% CI (1.10, 1.92), *P* < 0.01; I^2^ = 39%, τ^2^ = 0.00, Figure 8].

### Sensitivity analysis

To evaluate the robustness of our pooled findings, we conducted sequential leave-one-out sensitivity analyses. Given that Lu et al. (19) contributed the majority of the weight to the primary PONV analysis, we specifically excluded this trial. After removal, the pooled RR remained significant [RR = 0.44, 95% CI (0.27, 0.73), *P* < 0.01; I^2^ = 0%]. In addition to the leave-one-out analysis, excluding the single trial with a 12-h PONV assessment window (13) did not change the result [RR = 0.62, 95% CI (0.49, 0.78), I^2^ = 0%]. Excluding any other single trial did not materially alter the direction or significance of the effect sizes for PONV, pain, or QoR, indicating our main findings are stable.

### Publication bias

**Figure 9:**
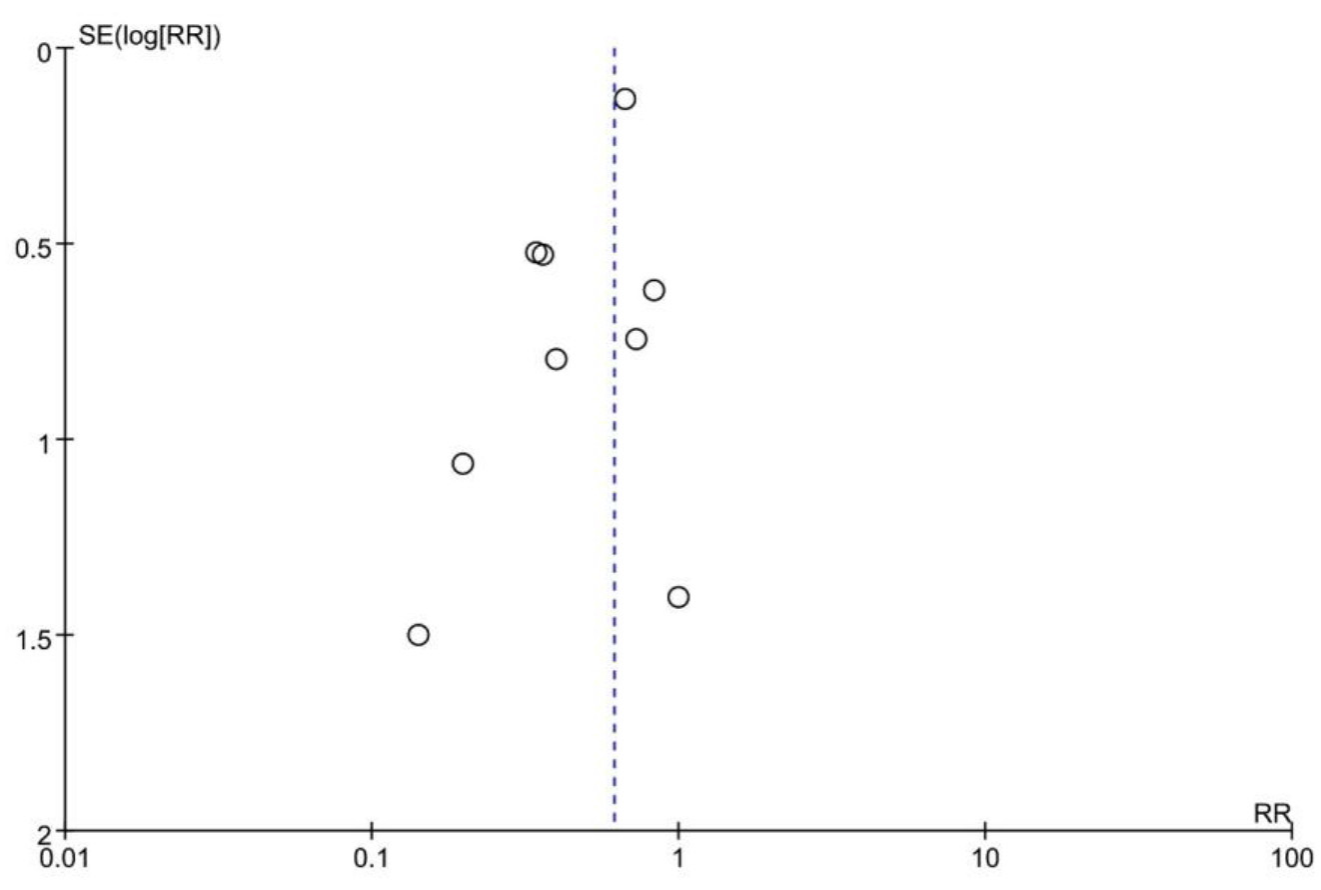
Funnel plot for the assessment of potential publication bias regarding the primary outcome (incidence of PONV).

A funnel plot was generated to visually assess potential publication bias for the primary outcome (Figure 9). Because fewer than 10 studies were included, formal statistical tests for funnel plot asymmetry (such as Egger's test) were not performed, as recommended by the Cochrane Handbook. Visual inspection of the funnel plot did not reveal obvious asymmetry, suggesting no clear publication bias. However, the funnel plot can only be interpreted qualitatively, and the small number of studies limits the power to detect asymmetry; thus, the possibility of publication bias cannot be entirely excluded.

### Certainty of evidence (GRADE)

Based on the GRADE framework, the overall certainty of evidence was adjusted to account for the methodological limitations of the included studies. The certainty of evidence for the primary outcome (reduction of overall PONV) was rated as “Low”. The absence of prospective trial registration in 13 of the 15 included studies raised concerns about selective outcome reporting, which contributed to the downgrading of evidence. This was downgraded due to the risk of bias for included trials and indirectness. The certainty of evidence for the secondary outcomes, including postoperative pain and QoR improvement, was rated as “Low.” These outcomes were downgraded by two levels: first for the aforementioned risk of bias, and second for inconsistency (due to the substantial clinical and statistical heterogeneity observed at later postoperative time points) and imprecision (due to the limited number of studies and smaller sample sizes reporting QoR scores). The summary result of GRADE was shown in Table 2.

**Table 2b:** GRADE evidence profile and certainty of evidence for primary and secondary outcomes.

Outcomes	No. of studies	Risk of bias	Inconsistency	Indirectness	Imprecision	Publication bias	Overall certainty
Incidence of overall PONV	9	Serious^a^	Not serious	Serious^b^	Not serious	Not serious	**Low** ⊕⊕⊖⊖
Incidence of PON	7	Serious^a^	Not serious	Serious^b^	Not serious	Not serious	**Low** ⊕⊕⊖⊖
Incidence of POV	7	Serious^a^	Not serious	Serious^b^	Not serious	Not serious	**Low** ⊕⊕⊖⊖
Requirement for rescue antiemetics	2	Serious^a^	Not serious	Not serious	Serious^c^	Not serious	**Low** ⊕⊕⊖⊖
Postoperative pain scores	Varies	Serious^a^	Serious^d^	Not serious	Not serious	Not serious	**Low** ⊕⊕⊖⊖
Early quality of recovery (QoR)	3	Serious^a^	Not serious	Not serious	Serious^e^	Not serious	**Low** ⊕⊕⊖⊖

GRADE, Grading of Recommendations Assessment, Development and Evaluation; PONV, postoperative nausea and vomiting; PON, postoperative nausea; POV, postoperative vomiting; QoR, Quality of recovery. ^**a**^Downgraded one level due to risk of bias: the majority of the included trials were assessed as having “some concerns”. ^**b**^All trials were conducted in China; the generalizability of findings to other populations or healthcare systems is uncertain. ^**c**^Downgraded one level due to imprecision: only two studies reported the requirement for rescue antiemetics, leading to a limited sample size. ^**d**^Downgraded one level due to inconsistency: substantial clinical and statistical heterogeneity was observed at later postoperative time points (12 and 24 h). ^**e**^Downgraded one level due to imprecision: limited number of studies and smaller sample sizes reporting QoR scores.

## Discussion

In this meta-analysis, we found that perioperative TEAS significantly reduced the incidence of PONV in patients undergoing breast cancer surgery. The benefits extended to isolated postoperative nausea and vomiting, reduced need for rescue antiemetics, and lower pain scores during the early postoperative period. Importantly, TEAS also improved early recovery quality, as measured by validated QoR questionnaires, without causing serious adverse events.

In the only prior TEAS meta-analysis that included mixed cancer surgeries (16 RCTs, 2,017 patients), the pooled RR for PONV was 0.47 (95% CI 0.37–0.61) (4). Our breast-specific pooled RR of 0.61 (95% CI 0.49–0.77) is directionally consistent but slightly higher, likely reflecting differences in baseline PONV risk and the higher proportion of sham-controlled trials in our analysis (three vs. an unknown proportion in the mixed-cancer review). Another meta-analysis of electrical acupoint stimulation (mostly non-cancer surgeries) reported a similar RR of 0.49 (27). These comparisons underscore that TEAS is broadly effective across surgical types, but breast surgery with its uniformly female population, volatile anesthetics, and postoperative opioids represents a particularly high-risk subgroup that deserves site-specific evidence (28).

Our findings extend those of Chen et al. (9), who reported that TEAS reduced PONV risk after general anesthesia across mixed surgical populations. Importantly, when restricted to the three sham-controlled trials (which were triple-blinded), TEAS still significantly reduced overall PONV, indicating that the observed antiemetic effect cannot be solely attributed to expectation or placebo bias. Furthermore, the present study showed that TEAS significantly improved QoR, relieved postoperative pain, and reduced rescue antiemetic use. Although Lu et al. (19) contributes the majority of weight, sensitivity analysis without this study shows similar trend, supporting robustness of results. Notably, the antiemetic effect of TEAS in our analysis was highly consistent, yielding zero statistical heterogeneity. The antiemetic and analgesic effects of TEAS are thought to involve vagal modulation and endogenous opioid release (29–31). This multi-target prophylactic mechanism parallels the efficacy of pharmacological antiemetics but avoids their associated adverse effects, such as QT interval prolongation or extrapyramidal symptoms (32). The reduction in PONV risk with TEAS is clinically meaningful and comparable to that of single-dose ondansetron or dexamethasone (33, 34). A recent non-inferiority trial by Zhang et al. demonstrated that TEAS combined with dexamethasone was non-inferior to granisetron plus dexamethasone for PONV prophylaxis in breast surgery (2). Unlike these drugs, TEAS carries no risk of QT prolongation, extrapyramidal symptoms, or sedation, making it especially valuable for patients with contraindications to standard antiemetics (14). Given the low certainty of evidence, TEAS should be viewed as a complementary adjunct within multimodal PONV prophylaxis, particularly for patients at high risk. For low- to moderate-risk patients, TEAS might serve as an alternative to a single pharmacologic antiemetic, but current evidence does not support TEAS as a standalone replacement for guideline-recommended antiemetics. Therefore, TEAS is best integrated into perioperative care as part of a multimodal strategy, not as a substitute for established pharmacologic prophylaxis.

TEAS has been reported to reduce postoperative pain through modulation of both peripheral and central pathways. It may activate Aβ and Aδ fibers, leading to inhibition of nociceptive transmission at the spinal dorsal horn (gate control theory). Centrally, TEAS may influence the periaqueductal gray–rostroventral medulla (PAG-RVM) pathway and promote endogenous opioid release. These mechanisms are consistent with our meta-analytic findings showing significant reductions in postoperative pain scores and analgesic consumption in breast surgery patients (35, 36). Another novel finding of our analysis is the temporal variation in statistical heterogeneity regarding pain scores. During the early postoperative phase (2, 4, and 6 h), the analgesic effect of TEAS was uniform across all included trials. This consistency likely reflects the direct, lingering neuromodulatory effects of intraoperative TEAS combined with highly standardized post-anesthesia care unit protocols. Conversely, substantial heterogeneity emerged at the 12 and 24 h marks. This divergence is clinically sound and expected, as it mirrors the transition from standardized early anesthesia care to ward-based management, where multimodal analgesia protocols, patient-controlled analgesia usage, and rescue analgesic thresholds vary significantly across different institutions.

The integration of TEAS aligns with the core tenets of ERAS. Traditional reliance on opioids for postoperative pain control often exacerbates PONV, creating a vicious cycle that delays ambulation and hospital discharge. By simultaneously alleviating pain and reducing nausea, TEAS facilitates an earlier return to normal physiological function. This holistic benefit is quantitatively reflected in the significantly improved QoR scores.

This meta-analysis has several limitations that warrant consideration. First, most included trials were evaluated as having “some concerns” regarding the risk of bias. Moreover, PONV, pain, and QoR are patient-reported outcomes that are inherently subjective and susceptible to bias. Second, the specific TEAS parameters, such as stimulation frequency, electrical intensity, intervention timing, and acupoint combinations, varied considerably across studies, precluding the recommendation of a single, standardized clinical protocol. Third, the vast majority of the included RCTs were conducted in China. Because genetic susceptibilities to PONV, baseline emetic risks, and cultural acceptance of acupuncture-related interventions may differ significantly across ethnicities, the generalizability (external validity) of our findings to Western populations or other global healthcare systems remains uncertain. Fourth, the limited number of sham-controlled studies precluded subgroup analyses for other outcomes, such as pain score. Fifth, breast-conserving surgery and mastectomy were both included, but the limited data precluded subgroup analysis by surgical extent. Additionally, most included trials lacked prospective trial registration. Although we observed no clear selective outcome reporting, this practice increases the risk of unreported outcomes and *post-hoc* analysis, which further lowers confidence in the effect estimates. Finally, critical secondary outcomes, including QoR scores and rescue antiemetic requirements, were reported by only a limited number of trials, restricting the statistical power for these endpoints.

## Conclusion

Based on low certainty evidence (GRADE), perioperative TEAS might be an effective adjunct for reducing PONV, alleviating postoperative pain, and enhancing early recovery in patients undergoing breast cancer surgery. Its non-invasive nature and favorable safety profile support its integration into perioperative care as part of multimodal strategies, particularly within ERAS pathways. Future research should focus on multicenter, sham-controlled RCTs to determine standardized stimulation parameters and confirm the observed benefits across diverse patient populations.

## Data Availability

The original contributions presented in the study are included in the article/Supplementary material, further inquiries can be directed to the corresponding authors.
