# Supplementary material for: Transcutaneous electrical acupoint stimulation for preventing postoperative nausea and vomiting in patients undergoing breast surgery: a systematic review and meta-analysis of randomized controlled trials
Source: Front Med (Lausanne). 2026 Jun 3;13:1830545. doi: 10.3389/fmed.2026.1830545 (PMC13271961; doi:10.3389/fmed.2026.1830545)

**Detailed Search Strategies**

**Search Date:** Up to March 10, 2026

**Database: PubMed** #1 "Breast Neoplasms"[Mesh] OR "breast cancer"[Title/Abstract] OR "breast neoplasm*"[Title/Abstract] OR "breast tumor*"[Title/Abstract] OR "breast surgery"[Title/Abstract] OR "mastectomy"[Title/Abstract] #2 "Transcutaneous Electric Nerve Stimulation"[Mesh] OR "Electroacupuncture"[Mesh] OR "Acupuncture Therapy"[Mesh] OR "transcutaneous electrical acupoint stimulation"[Title/Abstract] OR "TEAS"[Title/Abstract] OR "acupoint stimulation"[Title/Abstract] OR "electro-acupuncture"[Title/Abstract] OR "electroacupuncture"[Title/Abstract] #3 "Postoperative Nausea and Vomiting"[Mesh] OR "Nausea"[Mesh] OR "Vomiting"[Mesh] OR "postoperative nausea and vomiting"[Title/Abstract] OR "postoperative nausea"[Title/Abstract] OR "postoperative vomiting"[Title/Abstract] OR "PONV"[Title/Abstract] OR "nausea"[Title/Abstract] OR "vomiting"[Title/Abstract] #4 #1 AND #2 AND #3

**Database: Embase** #1 'breast tumor'/exp OR 'breast cancer':ab,ti OR 'breast neoplasm*':ab,ti OR 'breast tumor*':ab,ti OR 'breast surgery':ab,ti OR 'mastectomy':ab,ti #2 'transcutaneous nerve stimulation'/exp OR 'electroacupuncture'/exp OR 'acupuncture'/exp OR 'transcutaneous electrical acupoint stimulation':ab,ti OR 'TEAS':ab,ti OR 'acupoint stimulation':ab,ti OR 'electro-acupuncture':ab,ti #3 'postoperative nausea and vomiting'/exp OR 'nausea'/exp OR 'vomiting'/exp OR 'postoperative nausea and vomiting':ab,ti OR 'postoperative nausea':ab,ti OR 'postoperative vomiting':ab,ti OR 'PONV':ab,ti OR 'nausea':ab,ti OR 'vomiting':ab,ti #4 #1 AND #2 AND #3

**Database: The Cochrane Library (CENTRAL)** #1 MeSH descriptor: [Breast Neoplasms] explode all trees #2 ("breast cancer" OR "breast neoplasm*" OR "breast tumor*" OR "breast surgery" OR "mastectomy"):ti,ab,kw #3 #1 OR #2 #4 MeSH descriptor: [Transcutaneous Electric Nerve Stimulation] explode all trees #5 MeSH descriptor: [Electroacupuncture] explode all trees #6 ("transcutaneous electrical acupoint stimulation" OR "TEAS" OR "acupoint stimulation" OR "electroacupuncture"):ti,ab,kw #7 #4 OR #5 OR #6 #8 MeSH descriptor: [Postoperative Nausea and Vomiting] explode all trees #9 ("postoperative nausea and vomiting" OR "PONV" OR "nausea" OR "vomiting"):ti,ab,kw #10 #8 OR #9 #11 #3 AND #7 AND #10

**Database: Web of Science (Core Collection)** #1 TS=("breast cancer" OR "breast neoplasm*" OR "breast tumor*" OR "breast surgery" OR "mastectomy") #2 TS=("transcutaneous electrical acupoint stimulation" OR "TEAS" OR "acupoint stimulation" OR "electro-acupuncture" OR "electroacupuncture") #3 TS=("postoperative nausea and vomiting" OR "PONV" OR "postoperative nausea" OR "postoperative vomiting" OR "nausea" OR "vomiting") #4 #1 AND #2 AND #3

**Databases: Chinese Databases (CNKI, Wanfang, VIP)** #1 SU=("乳腺癌" OR "乳腺肿瘤" OR "乳腺手术" OR "乳房切除术") #2 SU=("经皮穴位电刺激" OR "穴位电刺激" OR "TEAS" OR "电针" OR "针刺") #3 SU=("术后恶心呕吐" OR "PONV" OR "恶心" OR "呕吐") #4 #1 AND #2 AND #3

**Supplement Figure.** Subgroup analysis of overall PONV incidence stratified by control type (sham‑controlled vs routine care).


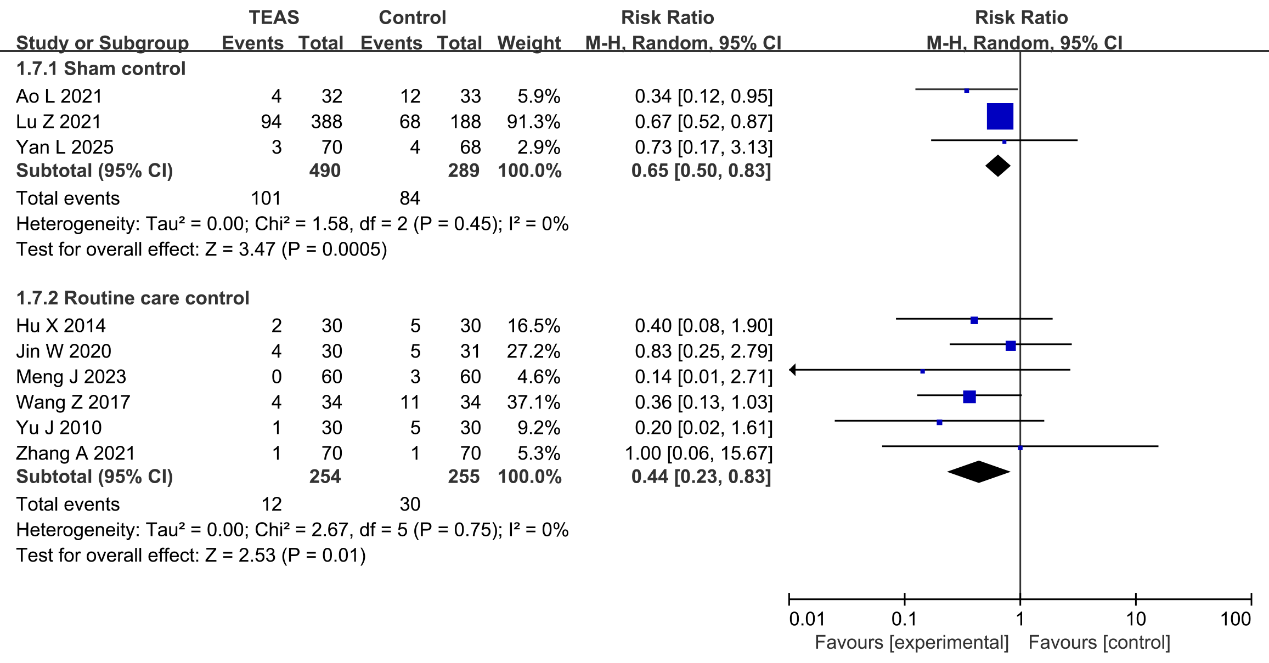

Supplement: Supplementary file 1 [file Table_1.docx]
